# Nurses’ willingness to care for patients infected with HIV or Hepatitis B / C in Vietnam

**DOI:** 10.1186/s12199-017-0614-y

**Published:** 2017-03-16

**Authors:** Tomohiro Ishimaru, Koji Wada, Huong Thi Xuan Hoang, Anh Thi My Bui, Hung Dinh Nguyen, Hung Le, Derek R. Smith

**Affiliations:** 1 Department of Occupational Health and Safety, Faculty of Public Health, Mahidol University, Bangkok, Thailand; 2 Occupational Health Training Center, University of Occupational and Environmental Health, Kitakyushu, Japan; 3 Bureau of International Health Cooperation, National Center for Global Health and Medicine, Tokyo, Japan; 4 Department of Infection Control, Faculty of Nursing, Thanh Tay University, Hanoi, Vietnam; 5 Department of Hospital Management, Health Management Training Institute, Hanoi University of Public Health, Hanoi, Vietnam; 6 Neurosurgical Department, Saint Paul Hospital, Hanoi, Vietnam; 7 Dong Da Hospital, Hanoi, Vietnam; 8 College of Public Health, Medical and Veterinary Sciences, James Cook University, Townsville, Australia

**Keywords:** Hepatitis B, Hepatitis C, Human immunodeficiency virus, Nurse, Stigma

## Abstract

**Objectives:**

This study examined the factors associated with nurses’ willingness to care for patients infected with human immunodeficiency virus (HIV) or hepatitis B or C virus (HBV/HCV) in Vietnam.

**Methods:**

A cross-section of 400 Vietnamese nurses from two hospitals were selected using stratified random sampling, to whom a self-administered questionnaire was administered which included demographic items, previous experience with patients infected with HIV or HBV/HCV, and their attitudes toward these patients. Data was analyzed using descriptive statistics and multiple logistic regression.

**Results:**

The lifetime prevalence of needlestick or sharps injury whilst caring for a patient infected with HIV or HBV/HCV was 9 and 15.8%, respectively. The majority of participants expressed a willingness to care for patients infected with HIV (55.8%) or HBV/HCV (73.3%). Willingness to care for HIV-infected patients was positively associated with being 40–49 years of age and confidence in protecting themselves against infection. Regarding HBV/HCV infection, willingness to care was positively associated with individual confidence in protecting themselves against infection.

**Conclusions:**

This study revealed that Vietnamese nurses were somewhat willing to care for patients infected with HIV or HBV/HCV, and this was associated with individual confidence in protecting themselves against infection and with negative attitudes towards HIV and HBV/HCV. Establishing a positive safety culture and providing appropriate professional education to help reduce the stigma towards infected patients offers an effective way forwards to improve quality of care in Vietnam, as elsewhere.

## Introduction

In Vietnam, patients infected with blood-borne diseases have been known to suffer from stigma and discrimination by healthcare workers [1], which is alarming given that the national prevalence of infectious disease is estimated to be 12.0% for *hepatitis B virus* (HBV), 2.0% for *hepatitis C virus* (HCV), and 0.5% for *human immunodeficiency virus* (HIV) [2, 3]. The primary transmission routes in Vietnam are believed to be prenatal transmission for HBV [4], injectable drug use and hemodialysis for HCV [5], and injectable drug use and unsafe sexual contact for HIV [6]. Since 2006, the government has prohibited refusal of care or discriminatory treatment against any patient infected with HIV [7], yet most hospitals still lack a formal policy and hospital practices to eliminate stigma and discrimination on this issue [8, 9]. Previous studies have suggested that injectable drug use and commercial sex were still considered as “social evils” in Vietnamese society; and these social mores, in turn, have the effect of strongly stigmatizing attitudes of healthcare workers toward patients infected with HIV [10, 11].

Nurses sometimes hold negative attitudes toward patients infected with HIV [12–15], HBV or HCV [16–18]. Providing care for such patients may put health professionals at risk for acquiring an infection, with the risk following a contaminated needlestick or sharps injury being estimated at between 6 and 30% for HBV, 1.8% for HCV, and 0.3% for HIV [19]. Although HBV infection can largely be prevented by vaccination; there is currently no effective vaccine for HIV or HCV, and a lack of effective post-exposure prophylaxis for HCV [20]. As such, risk perception may influence nurses’ unwillingness or refusal to care for patients with blood-borne diseases [21].

Positive attitudes toward patients infected with HIV or HBV/HCV represent an essential element in the appropriate care of such individuals. Nevertheless, few studies have investigated the factors affecting attitudes of Vietnamese nurses toward patients infected with HIV or HBV/HCV. This study was designed therefore, to examine the factors associated with nurses’ willingness to care for patients infected with HIV or HBV/HCV in Vietnam.

## Materials and methods

### Participants and procedure

We conducted a questionnaire study of Vietnamese nurses in February 2016. According to a report from the Vietnam Ministry of Health in 2008, 63,040 nurses were registered in Vietnam and were distributed as follows: 13% in central hospitals, 44% in province hospitals, 27% in district hospitals, and 17% in community hospitals [22]. Nurses in Vietnam are recognized in three categories depending on education levels (secondary educated nurses, college educated nurses and bachelor degree nurses). Secondary medical schools provide 2-years of training for nurses, junior colleges provide 3-year training for nurses, and universities provide a 4-year bachelor of nursing degree. All courses require 12-years of compulsory education for admission. Regardless of the different levels of their nursing education, however, all nurses work at the same level and scope in the clinical environment. The role of each nursing degree are currently under examination in Vietnam.

The target population for this study was nurses from two general hospitals located in Hanoi, Vietnam where patients infected with HIV, HBV or HCV were routinely cared for. Hospital A was 570 beds along with 500 nurses and Hospital B was 240 beds along with 300 nurses, respectively. Stratified random sampling was employed, with recruitment ceasing when 400 participants had joined the study (comprising 250 from Hospital A and 150 participants from Hospital B). The study questionnaire was completed anonymously and participants gave their written and oral informed consent. Each participant was paid 30,000 VND (approximately 1.5 USD) as a financial reward. The study was approved by the Hanoi School of Public Health Institutional Review Board (No. 016-004/DD-YTCC).

### Questionnaire

The questionnaire used for this study was adapted from previous research that had been undertaken in Japan [16]. It was translated into Vietnamese using standard translation procedures for cross-cultural studies [23, 24], with some items being modified for the local context following consultation with a panel of experts [25]. We collected information on demographics (gender, age, marital status, nurse category, and career duration) as well as previous experience caring for HIV or HBV/HCV infected patients, measured with binary yes/no responses to two questions: (1): professional contact with an infected patient within the previous year, and (2): previous needlestick or sharps injury (ever) whilst caring for a patient infected with HIV or HBV/HCV. Attitudes toward patients infected with HIV or HBV/HCV were examined using the following questions: “confidence to protect against infection during caring for an infected patient” (confidence); “avoid going near an infected patient” (discrimination); and “stigma that an infected patient is linked to homosexuality, injectable drug use, or having multiple sex partners” (stigma). Attitudes and willingness were assessed with two separate sets of questions, one related to HIV and the other to HBV/HCV. The dependent variable in this study was willingness to care for patients infected with HIV or HBV/HCV, and was measured as the participants’ level of agreement with the following statement: “I would want to care for a patient who is infected with HIV (or HBV/HCV)”. Each statement regarding attitudes or willingness to care was answered on a four-point Likert response scale (agree; somewhat agree; somewhat disagree; disagree).

### Data analysis

All variables were analyzed using descriptive statistics, with multiple logistic regression analysis being used to examine factors associated with nurses’ willingness to care for patients infected with HIV or HBV/HCV. Attitudes toward and willingness to care for, patients with HIV or HBV/HCV were treated as separate outcomes. The multivariable model included gender, age, marital status, nurse category, working experience, professional contact with an infected patient, and previous accidental injection or exposure to a patient with HIV or HBV/HCV. For the statistical analysis, attitudes toward patients infected with HIV or HBV/HCV were reclassified into three levels (1 = agree, 2 = somewhat agree, and 3 = disagree/somewhat disagree), and the outcomes were reclassified into two levels (1 = agree/somewhat agree, and 2 = disagree/somewhat disagree). We applied Zhang’s formula for adjusting the results of common outcomes [26]. All data were analyzed using SPSS for Windows 17.0 (SPSS Inc., Chicago, IL, USA), with p values <0.05 interpreted as being statistically significant.

## Results

A total of 400 nurses participated in the study, among whom, the majority were female, aged 20–39 years, married, and Secondary educated nurses (Table 1). Around half of participants (46.8%) had cared for patients infected with HIV in the past year, while 71.0% had cared for patients infected with HBV/HCV during this time period. In their professional experience as a nurse, 9 and 15.8% reported experiencing a previous needlestick or sharps injury while caring for a patient infected with HIV or HBV/HCV, respectively. Table 2 shows the frequency and distribution of attitudes toward patients infected with HIV or HBV/HCV. The majority answered “agree” or “somewhat agree” in response to the question of willingness to care for patients infected with HIV (55.8%) and HBV/HCV (73.3%). Approximately 70% of participants agreed or somewhat agreed that they felt confident to protect themselves from infection while caring for patients infected with HIV and HBV/HCV. While the majority of participants reported non-discriminatory and non-stigmatizing attitudes, some agreed or somewhat agreed that they still avoided going near patients infected with HIV (23.3%) and HBV/HCV (9.4%). In addition, some participants agreed with the statement that linked infected persons to homosexuality, injectable drug use, or having multiple sex partners (32.0% for HIV, 18.6% for HBV/HCV, respectively).

**Table 1:** Participant Demographics (*n* = 400)

	Number	Percent
Gender		
Male	56	(14.0)
Female	344	(86.0)
Age (years)		
20–29	111	(27.8)
30–39	189	(47.2)
40–49	57	(14.2)
50 and over	43	(10.8)
Marital status		
Married	322	(80.5)
Single	68	(17.0)
Divorced/widowed	10	(2.5)
Nurses’ education levels		
Secondary nurse	283	(70.8)
College nurse	44	(11.0)
Bachelor degree nurse	73	(18.2)
Professional experience (years)		
0–4	95	(23.8)
5–9	94	(23.5)
10–19	145	(36.2)
20 and over	66	(16.5)
Professional contact with patients infected with HIV or HBV/HCV(within the past year)		
HIV	187	(46.8)
HBV/HCV	284	(71.0)
Previous needlestick or sharps injury (ever) whilst caring for a patient infected with HIV or HBV/HCV		
HIV	36	(9.0)
HBV/HCV	63	(15.8)

*HIV*, Human Immunodeficiency, *HBV* Hepatitis B Virus, *HCV* Hepatitis C Virus

**Table 2:** Attitudes Toward Patients Infected with HIV or HBV/HCV (*n* = 400)

	HIV		HBV/HCV	
	n	(%)	n	(%)
Willingness to care for patients infected with HIV or HBV/HCV				
Agree	63	(15.8)	118	(29.5)
Somewhat agree	160	(40.0)	175	(43.8)
Somewhat disagree	74	(18.5)	54	(13.5)
Disagree	103	(25.7)	53	(13.2)
Confidence to protect myself from infection when caring for patients infected with HIV or HBV/HCV				
Agree	132	(33.0)	151	(37.8)
Somewhat agree	149	(37.2)	147	(36.7)
Somewhat disagree	51	(12.8)	47	(11.7)
Disagree	68	(17.0)	55	(13.8)
Avoid going near patients infected with HIV or HBV/HCV				
Agree	26	(6.5)	13	(3.2)
Somewhat agree	67	(16.8)	25	(6.2)
Somewhat disagree	62	(15.5)	43	(10.8)
Disagree	245	(61.2)	319	(79.8)
Do you believe that patients infected with HIV or HBV/HCV might be linked to homosexuality, injectable drug use, or have multiple sex partners?				
Agree	49	(12.2)	27	(6.8)
Somewhat agree	79	(19.8)	47	(11.8)
Somewhat disagree	61	(15.2)	45	(11.2)
Disagree	211	(52.8)	281	(70.2)

*HIV* Human Immunodeficiency, *HBV* Hepatitis B Virus, *HCV* Hepatitis C Virus

Multivariate analyses revealed factors associated with willingness to care for patients infected with HIV or HBV/HCV (Table 3). Regarding HIV infection, willingness to care for patients was positively associated with being 40–49 years old (*Odds Ratio* (OR) 1.70, *95% Confidence Interval* (95%CI): 1.02–2.07), and confidence in protecting myself against infection (agree: OR 1.97, 95%CI: 1.76–2.10; somewhat agree: OR 1.75, 95%CI: 1.48–1.94), and negatively associated with avoiding infected patients (agree: OR: 0.27, 95%CI: 0.10–0.66; somewhat agree: OR 0.61, 95%CI: 0.35–0.97) and being a bachelor degree trained nurse (OR: 0.62, 95%CI: 0.37–0.95). Regarding HBV/HCV infection, willingness to care for patients was only positively associated with confidence to protect against infection (agree: OR 2.27, 95%CI: 1.66–2.80; somewhat agree: OR 1.74, 95%CI: 1.21–2.30); willingness was negatively associated with the statement that linked infected persons to homosexuality, injectable drug use, or having multiple sex partners (agree: OR: 0.29, 95%CI: 0.12–0.66; somewhat agree: OR 0.33, 95%CI: 0.17–0.61) and with avoiding going near infected patients (somewhat agree: OR 0.34, 95%CI: 0.14–0.77).

**Table 3:** Factors associated with willingness to care for patients infected with HIV or HBV/HCV

	HIV				HBV/HCV			
	Univariate		Adjusted		Univariate		Adjusted	
	OR	(95% CI)	OR	(95% CI)	OR	(95% CI)	OR	(95% CI)
Age (years)								
20–29	1.00	-	1.00	-	1.00	-	1.00	-
30–39	0.90	(0.59–1.31)	1.27	(0.77–1.72)	1.06	(0.72–1.49)	1.56	(0.83–2.39)
40–49	2.09	(1.20–2.93)	1.70	(1.02–2.07)	1.28	(0.75–1.96)	2.21	(1.00–3.18)
50 and over	1.77	(0.94–2.68)	1.73	(0.91–2.13)	1.51	(0.83–2.31)	2.78	(1.28–3.52)
Confidence to protect myself from infection when caring for patients infected with HIV or HBV/HCV								
Disagree/somewhat disagree	1.00	-	1.00	-	1.00	-	1.00	-
Somewhat agree	1.80	(1.57–1.96)	1.75	(1.48–1.94)	1.53	(1.08–2.02)	1.74	(1.21–2.30)
Agree	2.03	(1.87–2.12)	1.97	(1.76–2.10)	2.36	(1.81–2.84)	2.27	(1.66–2.80)
Avoid going near patients infected with HIV or HBV/HCV								
Disagree/somewhat disagree	1.00	-	1.00	-	1.00	-	1.00	-
Somewhat agree	0.42	(0.26–0.64)	0.61	(0.35–0.97)	0.23	(0.10–0.49)	0.34	(0.14–0.77)
Agree	0.33	(0.15–0.67)	0.27	(0.10–0.66)	0.77	(0.27–1.73)	1.37	(0.48–2.60)
Do you believe that patients infected with HIV or HBV/HCV might be linked to homosexuality, injectable drug use, or have multiple sex partners?								
Disagree/somewhat disagree	1.00	-	1.00	-	1.00	-	1.00	-
Somewhat agree	0.55	(0.33–0.85)	0.70	(0.43–1.04)	0.32	(0.17–0.55)	0.33	(0.17–0.61)
Agree	0.55	(0.36–0.79)	0.90	(0.53–1.33)	0.27	(0.12–0.55)	0.29	(0.12–0.66)
Nurses’ education level								
Secondary nurse	1.00	-	1.00	-	1.00	-	1.00	-
College nurse	1.08	(0.73–1.44)	1.01	(0.62–1.43)	1.14	(0.64–1.81)	0.97	(0.50–1.67)
Bachelor degree nurse	0.70	(0.48–0.97)	0.62	(0.37–0.95)	0.82	(0.52–1.23)	0.79	(0.45–1.27)

*OR* Odds Ratio, *95%CI* 95% Confidence Interval, *HIV* Human Immunodeficiency Virus, *HBV* Hepatitis B Virus, *HCV* Hepatitis C Virus

## Discussion

This study investigated factors associated with nurses’ willingness to care for patients infected with HIV or HBV/HCV in Vietnam. Our study revealed that nurses who felt confident in protecting themselves against infection were more willing to care for patients infected with HIV or HBV/HCV, while nurses with discriminatory attitudes towards HIV and HBV/HCV, and stigma regarding HBV/HCV were less willing to care for such patients. The findings offer important insights for providing appropriate care for people infected with HIV, HBV or HCV in Vietnam, as elsewhere.

Healthcare workers may experience an ethical dilemma in deciding whether to provide treatment and care for patients infected with HIV, HBV or HCV. For example, unwillingness to treat patients with HIV has been reported in 23 to 50% of physicians in the United States, 21% in Spain and 14% in Canada [27]. The current study revealed that the percentages of Vietnamese nurses unwilling to provide care was similar to that reported among Japanese nurses (44% for HIV and 27% for HBV/HCV in Vietnam; 46% for HIV and 20% for HBV/HCV in Japan) [28]. Some studies have suggested that nurses may be more likely to give differential care to infected patients when compared to their medical counterparts [12, 29, 30], which may reflect relative differences in their knowledge of infection [31]. Nurses’ knowledge of infection is probably variable in Vietnam, due to the multiple education pathways to enter nursing, the lack of a national licensing examination, and the variety of practical training offered for newly graduated nurses. Our findings suggest that hospital managers should take action in this ethical dilemma among nurses so that infected patients can receive appropriate care.

Having a positive safety culture in the health care environment may improve nurses’ willingness to care for patients infected with blood-borne infections such as HIV, HBV or HCV. One study from Vietnam, for example, reported that nurses’ compliance with standard precautions was suboptimal, with 39% not washing their hands following patient contact [32]. Healthcare workers in such situations may fear cross-contamination, which may reduce their willingness to care for patients with blood-borne infections [33, 34]. The current study suggests that confidence in protecting oneself against infection was a positive factor associated with willingness to care for patients infected with HIV or HBV/HCV. In this regard, positive safety culture, such as strict infection control, may serve not only to protect healthcare workers but also to improve the quality of patient care [35].

Avoidant attitudes were negatively associated with willingness to care for patients infected with HIV and HBV/HCV in the current study. Such attitudes towards HIV, HBV and HCV are not uncommon in healthcare [36, 37]. In Vietnam, caring for HIV positive patients is often stigmatized due to the cultural contexts and historical events related to the disease; meaning that nurses often avoid going near infected patients for fear of suffering prejudice from colleagues and family members [9, 10, 38]. As such, it can be seen that greater efforts are clearly needed to improve the public image of patients with HIV, HBV and HCV infection. Community prejudice against individuals infected with blood-borne diseases is not limited to Vietnam however, and has also been reported in other Asian countries [39].

Our study suggests that stigma of nurses toward patients infected with HBV/HCV was negatively associated with willingness to care for them, while HIV stigma had no such link to willingness. In general, it can be suggested that individuals may harbor negative views toward HBV and HCV infection, although these are less stigmatized diseases when compared with HIV infection [34]. For example, some healthcare workers link patients infected with HCV to injectable drug use and uncooperative and problematic behavior in the wards [40–43]. Thus, HCV-related stigma might affect their unwillingness to provide care for such patients. Regarding HIV stigma, some previous studies have reported an association between homophobia and unwillingness to care for HIV patients [44], which is inconsistent with results from the current study. Further research is needed to clarify this inconsistency.

Middle-aged nurses may have more positive views about providing care for patients infected with HIV in Vietnam, with nurses aged 40–49 years being more likely to express willingness to care for such patients in the current study. On the other hand, a previous study from Japan reported that nurses aged 50 years or older were more stigmatized against HIV care, probably because they worked during the beginning of the AIDS epidemic [28]. Vietnam has a higher community HIV prevalence rate when compared to Japan (0.5% vs <0.1%) [45], and additional experiences with HIV care probably bring concomitant increases in knowledge and skills among middle-aged nurses; which may promote their willingness to care for infected patients [15, 46]. Although educational strategies clearly represent an appropriate strategy for reducing HIV stigma and discrimination in healthcare practice [47], our study suggests that the healthcare worker’s age must also be carefully considered.

Bachelor degree trained nurses were less willing to care for patients infected with HIV than secondary educated nurses in the current study. This result was inconsistent with previous research which focused on the length of education and professional license category (nurse or nursing aide) [30, 48]. Because the majority of nursing education in universities and colleges has been performed by physicians in Vietnam, the educators of physician tend to focus on medical management of diseases rather than nursing care, which might negatively affect nursing care for Vietnamese patients infected with HIV. As such, future research should examine nurse category-based differences in attitudes toward infected patients.

The current study incurred a few limitations which are worth considering. First, the study design was cross-sectional; and therefore, cause and effect relationships could not be determined. Additionally, we conducted the study in only two large public hospitals in Hanoi, and the sample was relatively small (*n* = 400). As these samples may not represent all nurses in Vietnam or elsewhere; our findings should be interpreted with caution.

In conclusion, this study revealed that nurses were somewhat willing to care for patients infected with HIV or HBV/HCV, and this willingness was associated with their confidence to protect themselves against infection, and with their discriminatory or stigmatizing attitudes toward groups of infected individuals. Establishing a positive safety culture and providing appropriate professional education to help reduce the stigma towards patients infected with HIV, HBV or HCV offers an effective way forwards to improve quality of care in Vietnam, as elsewhere.
